# O Que os Olhos Não Veem, o Coração Sente

**DOI:** 10.36660/abc.20220765

**Published:** 2023-07-21

**Authors:** Costantino Roberto Costantini, Costantino Ortiz Costantini, Sérgio Gustavo Tarbine, Marcos Antonio Denk, Vinicius Shibata Ferrari, Rafael Michel de Macedo

**Affiliations:** 1 Hospital Cardiológico Costantini Ltda Curitiba PR Brasil Hospital Cardiológico Costantini Ltda, Curitiba, PR – Brasil

**Keywords:** Ultrassom Intravascular, Ultrassom Intracoronário

Em dezembro de 1987, com mais de 300 implantes coronários experimentais realizados em cães e cadáveres, Palmaz e Schatz,^1^ acompanhados pelo Dr. Eduardo Sousa (Instituto Dante Pazzanese de Cardiologia), realizaram em São Paulo o primeiro implante de *stent* em um ser humano, com uma prótese desenvolvida pelo próprio Palmaz.^2^

No entanto, os resultados iniciais apresentados pelo primeiro estudo multicêntrico realizado sobre o tema não foram nada animadores, pois o dispositivo utilizado apresentou altos índices de oclusão subaguda (18%),^2^ apesar do *stent* de Palmaz apresentar inúmeros aspectos favoráveis de biocompatibilidade, de mecânica e de hemodinâmica.

Desta forma, com o intuito de diminuir os índices de oclusão subaguda, Schatz propôs uma alteração no desenho original do *stent* de Palmaz, sendo criado um novo modelo denominado Palmaz-Schatz. Além disso, para reduzir a relativa trombogenicidade da endoprótese, foi elaborado e aplicado um protocolo que previa a utilização de três fármacos antiagregantes associados a dois anticoagulantes, por via endovenosa e oral, iniciado imediatamente após o implante do *stent* .

Enquanto isso, paralelamente aos estudos conduzidos por Palmaz e Schatz (1987) para o tratamento percutâneo da doença arterial coronariana (DAC), outros autores, entre eles Paul Yock et al. (1988), apresentam, na *37th Annual Scientific Session of the American College of Cardiology* ,^3^ o primeiro estudo com resultados relacionados à utilização do ultrassom intracoronário (USIC) como uma ferramenta de excelente qualificação para o diagnóstico da DAC. Com essa nova tecnologia, seria possível avaliar a morfologia da superfície do vaso.

Com o avanço destes estudos, seria questão de tempo associar a realização do tratamento percutâneo à avaliação de imagem intravascular com o objetivo de visualizar, de forma efetiva e segura, a eficácia da técnica do implante de *Stent* . Isto de fato ocorreu em 1994, quando Nakamura e Goldenberg apresentaram ao mundo os primeiros resultados que relacionaram o procedimento de angioplastia transluminal coronariana (ATC) com a colocação de *stent* e a “nova” morfologia do vaso submetido ao procedimento visualizada a partir do USIC.^4^

A partir da apresentação dos resultados do trabalho de Nakamura et al.,^4^ os trabalhos anteriormente publicados passaram a ser questionados quanto às medidas utilizadas de desfecho de eficácia, pois o USIC demonstrou que a expansão do *stent* se demonstrava insuficiente na maioria dos casos (80%), a despeito do implante ter sido considerado bem sucedido, do ponto de vista angiográfico, de acordo com os critérios de momento. Assim sendo, os altos índices de trombose subaguda encontrados nos estudos iniciais, como o de Palmaz e Schatz,^4^ estariam relacionados à subexpansão de *stents* guiados somente por angiografia.^5^

A partir disto, foi desenvolvida a técnica da liberação ótima de *stents* guiada por USIC, descrita já no ano seguinte, em 1995, por Colombo et al.^6^ Esta técnica, associada à utilização de cateteres balão de alta pressão (> 12 atm), apresentou uma importante redução das taxas de trombose subaguda de 10-24% para 0,9%.^2 , 7 , 8^

A partir dos resultados deste estudo, foi descontinuado o uso de anticoagulação oral pela maioria dos serviços de cardiologia intervencionista, sendo substituída pela antiagregação plaquetária (ácido acetilsalicílico e tienopiridínicos), sendo simplificada, assim, a farmacoterapia adjunta, tanto no pré-tratamento como após o procedimento. Essa modificação resultou em uma redução no risco de sangramento com um maior efeito preventivo contra a trombose de *stent* , tendo como consequência um menor tempo de hospitalização dos pacientes.

Considerando esses achados e com o otimismo renovado, alguns intervencionistas começaram a empregar esta técnica rotineiramente em seus serviços, surgindo assim novos ensaios clínicos com séries maiores de pacientes. Ainda na era dos *Bare Metal Stents* (BMS), destacam-se duas metanálises que incluíram somente estudos randomizados, as quais demonstraram que a utilização rotineira do USIC reduziu a necessidade de novas revascularizações e eventos cardiovasculares adversos maiores (ECAM) quando comparada com a intervenção guiada somente por angiografia.^9 , 10^ Ao contrário destas evidências documentadas, o protocolo de um dos mais importantes estudos da era dos *stents* farmacológicos (SFs), o estudo Syntax I,^11^ utilizou o USIC para guiar o implante dos *stents* TAXUS em menos de 5% dos 903 pacientes randomizados; já o estudo Syntax II^12^ utilizou a ferramenta em 84,1% dos 450 pacientes randomizados, obtendo, assim, queda significativa nos índices de todas as revascularizações após um ano de seguimento em relação ao Syntax I (13,7% vs. 8,2%, respectivamente).

Diante das evidências científicas sobre os benefícios do uso sistemático do USIC para guiar e otimizar o implante dos SFs tanto de 1ª, 2ª e 3ª gerações em vários cenários clínicos distintos (lesões longas, lesões complexas, bifurcações, lesões de tronco de coronária esquerda e IAM), algumas metanálises demonstraram quedas significativas nos índices de infarto agudo do miocárdio (IAM), trombose (definitiva/provável), reestenose da lesão tratada, reestenose do vaso tratado, mortalidade cardiovascular e ECAM.^13 - 17^ Cabe lembrar que a angioplastia guiada por USIC contribuiu para o aprimoramento da ATC guiada por angiografia, uma vez que, ao longo dos anos, os conhecimentos adquiridos com o uso da imagem intravascular foram sendo adaptados para intervenções guiadas somente por angiografia, de modo que as intervenções que não utilizam USIC também apresentam elevado grau de segurança e eficácia.^18 - 19^

Embora exista atualmente um alto nível de confiança no valor clínico do uso do USIC para orientar e otimizar a intervenção coronária percutânea (ICP), a aplicação rotineira na prática diária do mundo real parece estar distante de ser uma realidade. Alguns dos motivos para esta baixa adesão foram relatados em um impactante artigo da revista *EUROINTERVENTION* publicado no ano de 2018, dos quais destacam-se alto custo (65,9%), aumento do tempo processual (35%), falta de reembolso (29,3%) e falta de treinamento para interpretar as imagens (17,1%).^20^

No entanto, tais barreiras só podem ser superadas pelo operador, o qual é o único responsável por garantir a implantação ótima de cada *stent* . Todavia, é de responsabilidade de toda a sociedade de cardiologia intervencionista transmitir esta mensagem aos seus jovens *fellows* , pois só assim o método será fortalecido. Precisamos incentivar a discussão sobre o atual nível de evidência científica relacionado à utilização do USIC, reavaliando as indicações atuais (IIa B). Caso contrário, teremos que conviver com as dúvidas do passado e também do presente em relação aos verdadeiros culpados pelas falhas dos *stents* . E, desta forma, editoriais como aqueles de Serruys et al.^21 , 22^ serão sempre atemporais: *“Who Is Thrombogenic: The Stent/Scaffold or the Doctor?”*

Diante deste cenário, entendemos que a frase atribuída a Confúcio, imperador e filósofo Chinês “Uma imagem vale mais que mil palavras” deveria ser um mandamento do intervencionismo, pois ao contrário do dito popular “O que os olhos não veem, o coração não sente”, a história do intervencionismo tem nos ensinado que: o que os nossos olhos não veem, o coração de muitos dos nossos pacientes sente.
