# Supplementary material for: O Que os Olhos Não Veem, o Coração Sente
Source: Arq Bras Cardiol. 2023 Jul 21;120(8):e20220765. [Article in Portuguese] doi: 10.36660/abc.20220765 (PMC10464859; doi:10.36660/abc.20220765)
Supplement: Translation

# Translation: Out of Sight, Out of Heart?

**Authors:** Costantino Roberto Costantini, Costantino Ortiz Costantini, Sérgio Gustavo Tarbine, Marcos Antonio Denk, Vinicius Shibata Ferrari, Rafael Michel de Macedo

**Affiliations:** 1 Brasil Hospital Cardiológico Costantini Ltda, Curitiba, PR – Brasil

**Keywords:** Intravascular Ultrasound, Intracoronary Ultrasound

In December 1987, with over 300 experimental coronary implants performed in dogs and cadavers, Palmaz and Schatz,^1^ accompanied by Dr. Eduardo Sousa (Dante Pazzanese Cardiology Institute), performed the first human stent implantation in São Paulo, with a prosthesis developed by Palmaz himself.^2^

However, the initial results of the first multicenter study on the subject were less than encouraging, as the device showed high rates of subacute occlusion (18%),^2^ despite the Palmaz stent presenting numerous favorable aspects of biocompatibility, mechanics, and hemodynamics.

Thus, aiming to reduce the rates of subacute occlusion, Schatz proposed a design modification to the original Palmaz stent, therefore creating a new model called the Palmaz-Schatz stent. Moreover, in order to reduce the relative thrombogenicity of the endoprosthesis, a protocol that provided for the use of three antiplatelet drugs associated with two anticoagulants, intravenously and orally, was designed and applied, starting immediately after stent implantation.

Meanwhile, in parallel with the studies conducted by Palmaz and Schatz (1987) for the percutaneous treatment of coronary artery disease (CAD), other authors, including Paul Yock’s et al. (1988), presented the first study related to the use of intracoronary ultrasound (ICUS) as an excellent qualification tool for the diagnosis of CAD at the 37th Annual Scientific Session of the American College of Cardiology.^3^ This new technology would allow for the evaluation of the vessel surface morphology.

With the advancement of these studies, it would be only a matter of time before percutaneous treatments were associated with intravascular image evaluation in order to effectively and safely visualize the efficacy of the stent implantation technique. Such an event actually occurred in 1994, when Nakamura and Goldenberg presented the first results relating to the procedure of transluminal coronary angioplasty (TCA) with the placement of a stent and the “new” vessel morphology submitted to ICUS visualization.^4^

From the presentation of Nakamura’s^4^ results, the previously published studies began to be questioned regarding their measures of efficacy endpoints, since the ICUS demonstrated that the expansion of the stent was proved insufficient in most cases (80%), despite the fact that the implant was considered successful from an angiographic standpoint, according to the criteria at the time. Thus, the high rates of subacute thrombosis found in initial studies, such as that of Palmaz and Schatz,^4^ would be related to the under expansion of stents guided by angiography only.^5^

This led to the development of an optimal stent release technique guided by ICUS, which was described in 1995 by Colombo et al.^6^ . This technique, associated with the use of high-pressure balloon catheters (> 12 atm), showed a significant reduction in subacute thrombosis rates from 10-24% to 0.9%.^2 , 7 , 8^

Based on the results of this study, oral anticoagulation therapy was discontinued by most interventional cardiology services, and replaced with antiplatelet therapy (acetylsalicylic acid and thienopyridines), thus simplifying adjunctive pharmacotherapy, both pre-treatment and after the procedure. This modification reduced the risk of bleeding with a greater preventive effect against stent thrombosis, resulting in shorter hospital stay for patients.

Considering these findings, some optimistic interventionists began to use this technique routinely in their services, thus giving rise to new clinical trials with larger series of patients. Still in the Bare Metal Stents (BMS) era, two meta-analyses stand out, which included randomized studies demonstrating that the routine use of ICUS reduced the need for new revascularizations and major adverse cardiovascular events (MACE) when compared with intervention guided exclusively by angiography.^9 , 10^ Contrary to this documented evidence, the protocol of one of the most important studies of the drug-eluting stents (DES) era, Syntax I,^11^ used ICUS to guide the implantation of TAXUS stents in less than 5% of the 903 randomized patients; the Syntax II study^12^ used it in 84.1% of the 450 randomized patients, thus obtaining a significant drop in all revascularizations rates after a one-year follow-up compared to Syntax I (13.7% vs. 8.2%, respectively).

Given the scientific evidence on the benefits of the systematic use of ICUS to guide and optimize the implantation of 1^st^, 2^nd^, and 3^rd^generation DES in several different clinical scenarios (long lesions, complex lesions, bifurcations, lesions of the left main coronary, and AMI), some meta-analyses have shown significantly decreased rates of acute myocardial infarction (AMI), thrombosis (definite/probable), restenosis of the treated lesion, restenosis of the treated vessel, cardiovascular mortality, and MACE.^13 - 17^ It is worth noting that ICUS-guided angioplasty has contributed to the improvement of angiography-guided TCA since the knowledge acquired over the years with the use of intravascular imaging has been adapted for interventions guided exclusively by angiography, so that interventions that do not use ICUS also have a high degree of safety and efficacy.^18 - 19^

Although there is currently a high level of confidence in the clinical value of using ICUS to guide and optimize percutaneous coronary intervention (PCI), routine real-world applications seem to be far from being a reality. Some of the reasons for this low adherence were reported in a breakthrough article published in 2018 in the EUROINTERVENTION journal, which points out to high cost (65.9%), increased procedural time (35%), lack of reimbursement (29.3%) and lack of image interpretation training (17.1%).^20^

However, such barriers can only be overcome by the operator, who is the sole responsible for ensuring the optimal implantation of each stent. However, it is the entire society of interventional cardiology’s responsibility to convey this message to its younger fellows, as this is the only way to actually strengthen the method. Discussion about the current level of scientific evidence related to the use of ICUS needs to be encouraged by reassessing current indications (IIa B). Otherwise, we will have to live with the doubts of the past and present regarding the real culprits for stent failures. Thus, editorials like those by Serruys and Colombo^21 , 22^ will always be timeless works: *“* Who Is Thrombogenic: The Stent/Scaffold or the Doctor?”

Given this scenario, the phrase attributed to Chinese emperor and philosopher Confucius “A picture is worth a thousand words” should be the leading commandment of interventionism, because contrary to the popular saying “out of sight, out of mind,” the history of interventionism has taught us that even what our patient’s eyes can’t see, their minds and hearts can surely feel.
